# Hypermethylation of heparanase 2 promotes colorectal cancer proliferation and is associated with poor prognosis

**DOI:** 10.1186/s12967-021-02770-0

**Published:** 2021-03-05

**Authors:** Hui Zhang, Chenxin Xu, Chen Shi, Junying Zhang, Ting Qian, Zhuo Wang, Rong Ma, Jianzhong Wu, Feng Jiang, Jifeng Feng

**Affiliations:** 1 Department of General Surgery, Jiangsu Cancer Hospital, Jiangsu Institute of Cancer Research, The Affiliated Cancer Hospital of Nanjing Medical University, Nanjing, 210000 Jiangsu People’s Republic of China; 2 Research Center for Clinical Oncology, Jiangsu Cancer Hospital, Jiangsu Institute of Cancer Research, The Affiliated Cancer Hospital of Nanjing Medical University, 42 Baiziting, Nanjing, 210000 Jiangsu People’s Republic of China; 3 Department of Oncology, Jiangsu Cancer Hospital, Jiangsu Institute of Cancer Research, The Affiliated Cancer Hospital of Nanjing Medical University, Nanjing, 210000 Jiangsu People’s Republic of China; 4 Jiangsu Key Laboratory of Molecular and Translational Cancer Research, Jiangsu Cancer Hospital, Jiangsu Institute of Cancer Research, The Affiliated Cancer Hospital of Nanjing Medical University, 42 Baiziting, Nanjing, 210000 Jiangsu People’s Republic of China

**Keywords:** Colorectal cancer, Heparanase 2, Methylation, Biomarker, Proliferation

## Abstract

**Background:**

The epigenetic abnormality of tumor-associated genes contributes to the pathogenesis of colorectal carcinoma (CRC). However, methylation in colorectal cancer is still poorly characterized.

**Method:**

By integration of DNA methylation data from the GEO database and gene expression data from The Cancer Genome Atlas database, the aberrantly methylated genes involved in CRC tumorigenesis were identified. Subsequent in vitro experiments further validated their role in CRC.

**Results:**

We performed integrative genomic analysis and identified HPSE2, a novel tumor suppressor gene that is frequently inactivated through promoter methylation in CRC. K-M survival analysis showed that hypermethylation–low expression of heparanase 2 (HPSE2) was related to poor patient prognosis. Overexpression of HPSE2 reduced cell proliferation in vivo and in vitro. HPSE2 could regulate the p53 signaling pathway to block the cell cycle in G1 phase.

**Conclusion:**

HPSE2, a novel tumor suppressor gene that is frequently inactivated through promoter methylation in CRC. HPSE2 performs a tumor suppressive function by activating the p53/ p21 signaling cascade. The promoter hypermethylation of HPSE2 is a potential therapeutic target in patients with CRC, especially those with late-stage CRC.

## Background

Colorectal cancer (CRC) remains the third most common cancer and the second leading cause of cancer deaths worldwide [1] despite dramatic drops in its overall incidence and mortality among people aged 50 and older in recent years [2].

The epigenetic abnormality of tumor pathogenic genes, particularly the DNA methylation of selected gene promoters, contributes to the pathogenesis of CRC [3, 4]. DNA methylation is a covalent DNA modification that converts the DNA base cytosine to 5-methylcytosine; this process is catalyzed by DNA methyltransferases [5]. Under physiological conditions, CpG island methylation actively regulates the balance between DNA methylation and DNA demethylation to maintain proper gene expression patterns [6]. Aberrantly methylated genes can promote the pathogenesis of CRC by regulating specific signaling pathways [7]. CpG island methylator phenotype (CIMP), a specific pattern of promoter methylation, may serve as a biomarker for early detection and as a tool for monitoring patients with CRC. DNA methylation alterations may also be useful tools for CRC diagnosis, prediction, and treatment [8, 9]. However, methylation in CRC remains poorly characterized. The development of The Cancer Genome Atlas Network (TCGA dataset) and other public databases has enabled comprehensively analyzing the molecular characteristics of cancer genomes, transcriptomes, epigenomics, and proteomes, thus opening up a new path for cancer molecular diagnosis [10, 11].

We previously screened two tumor suppressor genes with known functions that are regulated by methylation, which confirmed the reliability of our analysis [12]. To find novel pathogenic genes, we analyzed and identified 10 aberrantly methylated genes by integrating DNA methylation data from the GEO database and gene expression data from TCGA database. On the basis of subsequent Kaplan–Meier plot validation, we focused on heparanase 2 (HPSE2) whose hypermethylation–low expression was related to poor patient prognosis. HPSE2 overexpression reduced cell proliferation in vivo and in vitro. HPSE2 could regulate the p53/P21 signaling pathway to arrest the cell cycle in the G1 phase. Collectively, we performed integrative genomic analysis and identified HPSE2 as a tumor suppressor gene in CRC. Aberrantly expressed HPSE2 could promote tumor aggressiveness via the p53/p21 signaling pathway.

## Methods

### Screening of methylation-regulated genes from the GEO and TCGA databases

To screen for methylation-regulated genes, we counted and analyzed aberrantly methylated genes between tumors and adjacent normal tissues in GSE17648 and GSE29490 from the GEO database (https://www.ncbi.nlm.nih.gov/geo/). P < 0.05 and | logFC |≥ 1 were considered statistically significant. Correspondingly, genes that were abnormally expressed in CRC were screened from TCGA database (https://portal.gdc.cancer.gov/) (P < 0.05 and |log FC |≥ 3). By taking the intersection, we obtained genes showing hypermethylation–low expression and hypomethylation–high expression in tumor tissues. We performed Pearson correlation (cor) to evaluate the relationship between gene expression and methylation. Cor <  − 0.3 and P < 0.05 were considered significant and further analyzed. Kaplan–Meier plot survival analysis was conducted to evaluate the role of methylation-regulated genes in the prognosis of patients with CRC.

### Univariate and multivariate Cox regression analysis

Univariate and multivariate Cox proportional hazard regression analyses were performed to investigate the effects of various clinical features (age, gender, T stage, N stage and M stage) and HPSE2 on the OS of patients with CRC. The HR and 95% confidence interval were assessed. Multivariate Cox regression analysis was used to verify the independent predictive capacity of HPSE2 when compared with that of other clinical factors.

### CpG island methylation of HPSE2

The methylation data for the CpG islands of the HPSE2 promoter, which are located 3 kb upstream, were downloaded from TCGA database. We obtained 49 CpG islands and analyzed their correlation with gene expression. Cor <  − 0.3 and P < 0.05 was considered statistically significant and retained for further analysis.

### Sample collection

Sixty patients with pathologically confirmed CRC diagnosed within the past one year at the Affiliated Cancer Hospital of Nanjing Medical University (Nanjing, China) were enrolled in the study. Peripheral blood was obtained preoperatively from 45 patients with CRC. In addition, the peripheral blood samples of 44 healthy controls were collected from the Affiliated Geriatric Hospital of Nanjing Medical University in Nanjing, China. None of the patients received preoperative chemotherapy or radiotherapy. All the specimens were immediately frozen in tubes containing RNAlater preservation liquid after removal and stored at liquid nitrogen. This study was approved by the Ethics Boards of Jiangsu Cancer Hospital. Written informed consent was signed by each patient.

### Cell culture, stable cell line construction, and HPSE2-overexpression

Human CRC cell lines SW480, HCT116, SW620, LOVO, DLD1, and NCM460 were purchased form the American Type Culture Collection (Manassas, Virginia, USA). The cells were cultured at 37 °C in a 5% CO_2_ incubator in Dulbecco’s modified Eagle’s medium (KeyGEN BioTECH, Jiangsu, China) with 10% fetal bovine serum (Gibco, USA). Lentivirus-HPSE2 (LV-HPSE2) were constructed in the lentivirus vector GV492 by a commercial service (Genechem Biotech Inc, Shanghai, China), while lentivirus vector GV492 was adopted as the control. LV-HPSE2 was packaged in 293 T cells. The supernatant of cell culture medium containing lentivirus granules was collected and the viral titer of the virus solution was determined. HCT116 and SW480 with a growth fusion degree of about 80% were digested by trypsinase and the cell suspension was prepared. Cells were seeded into six-well plates at 1.0 × 10^6^ cells per well and maintained at 37℃ in a humidified atmosphere of 5% CO_2_. Cells were divided into two groups, one group was added with 10 ml virus solution and the other group was added with 10 ml empty vector virus solution. The multiplicity of infection (MOI_HCT116_ and MOI_SW480_) was 10. After 16 h of cell culture, the culture medium was replaced. After 3 days of infection, the cells were in good condition. The positive clones were screened by using a complete culture medium containing 2.0 mg/mL puromycin for 4 weeks.

### RNA extraction, reverse transcription and qRT-PCR

Tissue and blood RNA were extracted according to the instruction of Tissue RNA Kit (OMEGA bio-tek, R6688-01, USA) and Blood RNA Kit (OMEGA bio-tek, R6814-01C, USA). TRIzol reagent (Invitrogen) was utilized to extract RNAs from cultured cells according to manufacturer’s instructions. A ratio of (A260)/(A280) is an indication of nucleic acid purity. A value greater than 1.8 indicates > 90% nucleic acid purity. For qRT-PCR, 1 μg RNAs were inversely transcribed into 20 μl cDNA with a Reverse Transcription Kit (Takara, Dalian, China). qRT-PCR was analyzed according to our previously published article [12]. The relative expression of HPSE2 was performed for three independent times and normalized using the 2^− ΔΔCt^ method relative to GAPDH.

The primers were as follows: GAPDH-Forward: GGTGAAGGTCGGAGTCAACG,

GAPDH-Reverse: TGGGTGGAATCATATTGGAACA,

HPSE2-Forward: ATGGCCGGGCAGTAAATGG,

HPSE2-Reverse: GCTGGCTCTGGAATAAATCCG.

### Western blot analysis

Total protein was lysed with RIPA extraction reagent (ThermoFisher, USA) that contained with a protease inhibitor cocktail (Beyotime Biotechnology, China). Protein lysates were separated by 10% sodium dodecyl sulfate polyacrylamide gel electrophoresis (SDSP-AGE) and transferred to 0.22 mm polyvinylidene fluoride membranes (Millipore, USA). The membranes were subsequently blocked in 5% defatted milk and incubated with primary antibodies overnight at 4 °C. Specific bands were visualized by ECL chromogenic substrate and quantified by densitometry (Quantity One software, BioRad). The following antibodies were used: HPSE2 (1:500; #ab127204, abcam, Cambridge, UK), p53 (1:1000; #2527, Cell Signaling Technology, Inc., MA, USA), p21 (1:1000; #2947, Cell Signaling Technology, Inc., MA, USA), GAPDH (1:1000; #5174, Cell Signaling Technology, Inc., MA, USA), and ki67 (1:1000; #ab15580, abcam, Cambridge, UK).

### CCK8

Cell proliferation was evaluated by using a CCK-8 kit (Dojindo, Japan) in accordance with the manufacturer’s instructions. Briefly, 3 × 10^3^ cells were seeded in a 96-well plate, and 10 µl of CCK8 solution and 100 µl of DMEM were added to the culture for 1 h in the dark to detect absorbance at 450 mm.

### Transwell experiments

A total of 200 µl of cell suspension was diluted with serum-free medium and added to the upper culture dish of a Transwell chamber (8 mm well, Corning, USA) to ensure that the number of cells in the upper layer was not less than 5 × 10^4^. A total of 500 µl of medium containing 10% FBS was added to the lower culture dish. After 36 h of incubation at room temperature, the cell membrane was fixed with 4% paraformaldehyde, stained with crystal violet, and photographed under an inverted microscope.

### Cell cycle experiments

Cell counting experiments and cell cycle experiments were performed by using a cell cycle detection kit (KeyGEN BioTECH #KGA511-KGA512) in accordance with instructions.

### Xenograft mouse carcinogenesis model

All animal research procedures were approved by the Institutional Animal Care and Use Committee of Sun Yat-sen University. Twelve female 6-week-old BALB/cA-nude mice were purchased from Nanjing (Nanjing, China). A total of 1 × 10^6^ HCT116 cells were injected subcutaneously into the flanks of the mice. The weight of the mice was measured every week, and the size of subcutaneous tumors was observed. The animals were euthanized after 5 weeks, and tumor volume was measured (volume = 4/3pr^3^). Finally, tumor tissues were embedded, fixed, and prepared for IHC staining. Cancer tissue was cut into 6.0 mm sections and stained with anti-ki67 antibody for IHC. Images were captured by using an AxioVision Rel. 4.6 computer image analysis system (Carl Zeiss).

### GSEA enrichment analysis

We performed GSEA enrichment analysis with GSEA v4.0.3 (https://www.gsea-msigdb.org/gsea/downloads.jsp). The reference gene set of KEGG enrichment analysis was c2.cp.kegg.v7.2.symbols.gmt (http://www.gsea-msigdb.org/gsea/downloads.jsp). Gene expression data of 647 CRC samples were downloaded from TCGA database and classified into 2 groups (High HPSE2 expression group vs. Low HPSE2 expression group) by the median expression of HPSE2. Then, the rest steps were carried out according to the conventional analysis method of GSEA software [13, 14].

### Statistical analysis

RNA-seq data analysis was performed with R 3.6.0 software. The R packages “limma”, “survival”, “plyr”, “ggplot2”, “grid”, “gridExtra”, and “ggpubr” were used according to the instruction of bioconductor website (http://www.bioconductor.org/). All of the measurement data were expressed as mean ± SD. Univariate and multivariate Cox regression analysis were performed with SPSS 22 software (Chicago, IL, USA). GraphPad Prim 5 (GraphPad Software, La Jolla, USA) was applied to statistically analyze qPCR results. Student’s t-test was used to evaluate statistical differences between 2 groups. P < 0.05 was considered statistically significant.

## Results

### Screening of methylation-regulated genes from the GEO and TCGA databases

**Fig. 1:**
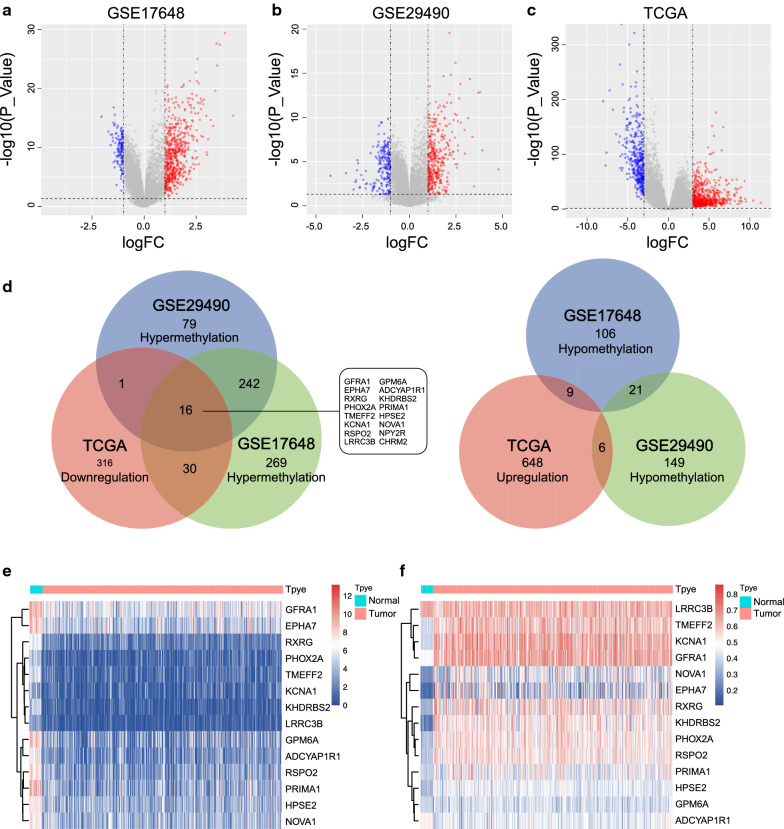
Identification of aberrantly methylation-regulated genes from GEO and TCGA databases. Aberrant DNA methylation between CRC tumors and adjacent normal tissues in GSE17648 (**a**) and GSE29490 (**b**); **c** Ectopically expressed genes in CRC tumor tissues from TCGA database; **d** Venn diagram of DNA methylation and expression in tumor tissue; **e**, **f** Heat map showing the differential expression and methylation of 14 genes between tumors and adjacent normal tissues from TCGA database

We selected 2 methylation datasets (GSE17648 and GSE29490) to screen for aberrantly methylation-regulated genes. In accordance with the criteria, 692 differentially methylated genes (136 hypermethylated and 557 hypomethylated genes) between tumors and normal tissues from the GSE17648 dataset (Fig. 1a and Additional file 1: Table S1) and 514 differentially methylated genes (176 hypermethylated and 338 hypomethylated genes) from the GSE29490 dataset were identified (Fig. 1b). Additionally, 1026 aberrantly expressed genes (663 upregulated and 363 downregulated genes) were obtained from TCGA database (Fig. 1c and Additional file 1: Table S1). Finally, sixteen hypermethylated–lowly expressed and 0 hypomethylated–highly expressed genes were screened out (Fig. 1d) by taking the intersection.

We mapped the methylation and expression data of 16 genes from TCGA database, which contained 407 CRC tissues and 21 adjacent tissues (Fig. 1e, f). NPYR and CHR2 were deleted due to the absence of corresponding methylation information.

**Fig. 2:**
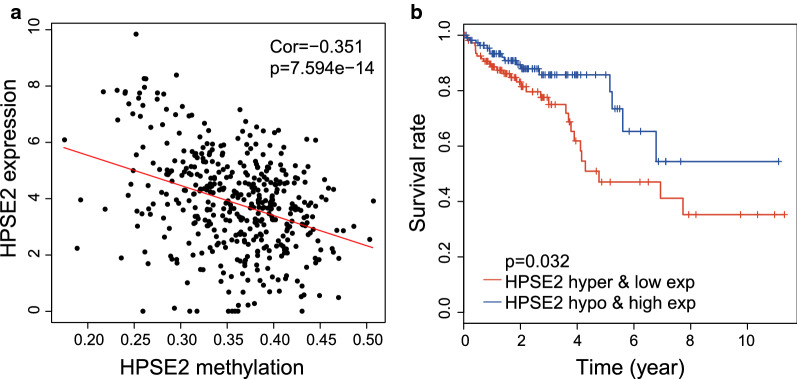
Expression and methylation of HPSE2 was associated with 5-year overall survival in CRC patients. **a** Pearson correlation between HPSE2 expression and methylation; **b** Patients with hypermethylation–low expression of HPSE2 have a worse prognosis

The Pearson correlation between expression and methylation was further evaluated, and 10 out of the 14 methylation-regulated genes were screened in accordance with the criteria (cor <  − 0.3, Table 1, Fig. 2a and Additional file 2: Fig. S1A-I). We considered that those genes might participate in the development of tumors and affect the prognosis of patients. Kaplan–Meier plot analysis was therefore performed and 403 patients with CRC were divided into hypomethylation–high expression (hypo and high exp) and hypermethylation–low expression (hyper and low exp) groups. As shown in Fig. 2b and Additional file 2: Fig S1J–R, only HPSE2 could be used to clearly distinguish the survival of patients. Patients with the hypermethylation–low expression of HPSE2 had significantly poor survival (P = 0.032).

**Table 1:** Correlation between gene expression and methylation of CRC in TCGA database

Gene	Methylation						Expression						Correlation	
	Normal			Tumor	P		Normal		Tumor		P		Cor	P
KHDRBS2	0.156 ± 0.031			0.443 ± 0.091	< 0.001		4.678 ± 0.981		0.753 ± 1.007		< 0.001		− 0.594	< 0.001
GPM6A	0.308 ± 0.150			0.325 ± 0.053	< 0.001		7.837 ± 1.658		3.036 ± 2.459		< 0.001		0.018	0.708
HPSE2	0.259 ± 0.022			0.363 ± 0.053	< 0.001		7.536 ± 0.506		3.660 ± 1.555		< 0.001		− 0.351	< 0.001
KCNA1	0.316 ± 0.027			0.557 ± 0.104	0.131		3.389 ± 1.915		1.096 ± 1.367		< 0.001		− 0.383	< 0.001
PHOX2A	0.270 ± 0.038			0.451 ± 0.075	< 0.001		4.470 ± 1.039		1.090 ± 1.259		< 0.001		− 0.411	< 0.001
RSPO2	0.283 ± 0.034			0.450 ± 0.075	< 0.001		7.715 ± 0.906		2.982 ± 1.805		< 0.001		− 0.455	< 0.001
RXRG	0.242 ± 0.137			0.461 ± 0.116	< 0.001		6.019 ± 0.590		1.920 ± 1.284		< 0.001		− 0.404	< 0.001
GFRA1	0.393 ± 0.017			0.571 ± 0.079	< 0.001		9.050 ± 0.826		5.791 ± 1.885		< 0.001		− 0.357	< 0.001
TMEFF2	0.324 ± 0.022			0.517 ± 0.089	< 0.001		5.336 ± 0.848		1.260 ± 1.291		< 0.001		− 0.431	< 0.001
LRRC3B	0.577 ± 0.026			0.534 ± 0.081	0.016		3.120 ± 0.969		0.667 ± 0.874		< 0.001		0.138	0.004
NOVA1	0.175 ± 0.130			0.359 ± 0.118	< 0.001		0.232 ± 0.016		0.437 ± 0.127		< 0.001		− 0.492	< 0.001
ADCYAP1R1	0.431 ± 0.010			0.359 ± 0.059	< 0.001		6.537 ± 1.170		2.442 ± 1.826		< 0.001		0.246	< 0.001
PRIMA1	0.301 ± 0.015			0.390 ± 0.093	< 0.001		9.030 ± 1.064		3.451 ± 2.171		< 0.001		− 0.222	< 0.001
EPHA7	0.121 ± 0.009			0.340 ± 0.155	< 0.001		8.333 ± 1.425		4.669 ± 1.993		< 0.001		− 0.431	< 0.001

### Hypermethylation-low expression of HPSE2 was an independent predictor of the poor outcome of patients with CRC

**Fig. 3:**
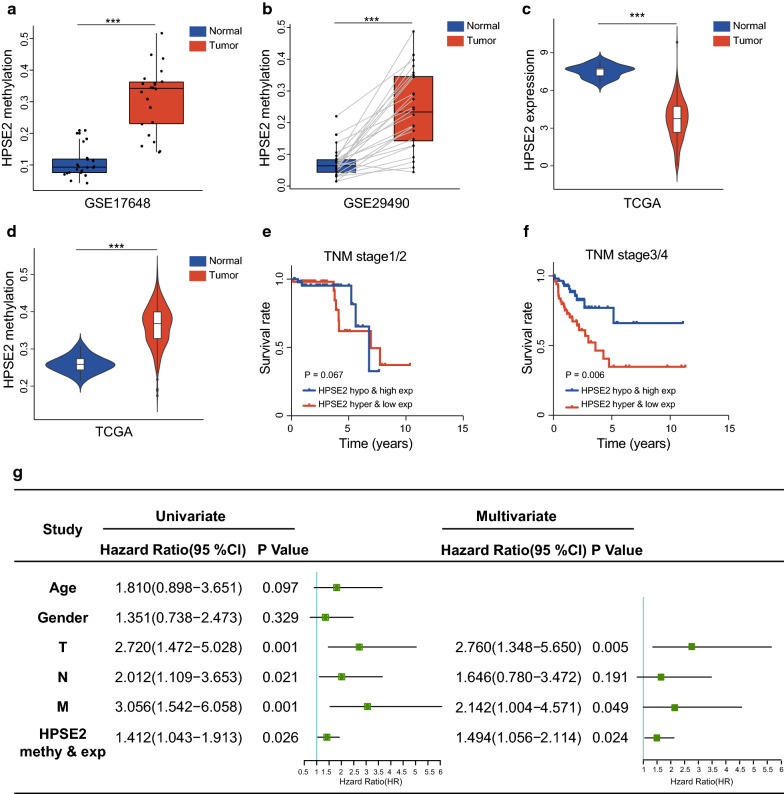
HPSE2, which was regulated by promoter DNA methylation, could be an independent prognostic factor. **a** Methylation levels of HPSE2 in 22 cases of CRC tissues in GSE17648; **b** Methylation levels of HPSE2 in 26 cases of matched CRC tumors and adjacent normal tissues in GSE29490; HPSE2 expression (**c**) and methylation (**d**) in TCGA database; **e**, **f** Kaplan–Meier curves showing that hypermethylation–low expression of HPSE2 was associated with the shortened survival of CRC patients at the late stages (**f**) but not at the early stages (**e**); **g** Univariate and multivariate COX regression model revealed that HPSE2 could be an independent prognostic risk factor. ***P < 0.001

HPSE2 was significantly abnormally methylated (GSE17648 and GSE29490) and expressed in tumor tissue (TCGA database) as shown in Fig. 3a–d. Then, patients with CRC were divided into the hypermethylation-low expression and hypomethylation-high expression groups in accordance with the median of HPSE2 (the expression level was 3.660 and the methylation level was 0.367). Further stratification by TNM stage revealed that patients with HPES2 hypermethylation had significantly shortened survival in stage III/IV (P < 0.05) but not in stages I/II (P > 0.05; Fig. 3e, f). Univariate Cox regression revealed that HPES2 hypermethylation–low expression was associated with an increased risk of cancer-related death (relative risk [RR]:1.412; 95% CI 1.043–1.913, P = 0.026). As expected, T-N-M stage was also a significant prognostic factor. In particular, multivariate Cox regression analysis revealed that HPSE2 methylation–expression was an independent risk factor for shortened survival among patients with CRC (RR: 1.494; 95% CI 1.056 to 2.114, P = 0.024) (Fig. 3g). These findings indicated that the hypermethylation–expression of HPSE2 was predictive of the poor prognosis of patients with CRC, especially those in the late stages of the disease.

### CpG islands methylation of HPSE2 in CRC

**Fig. 4:**
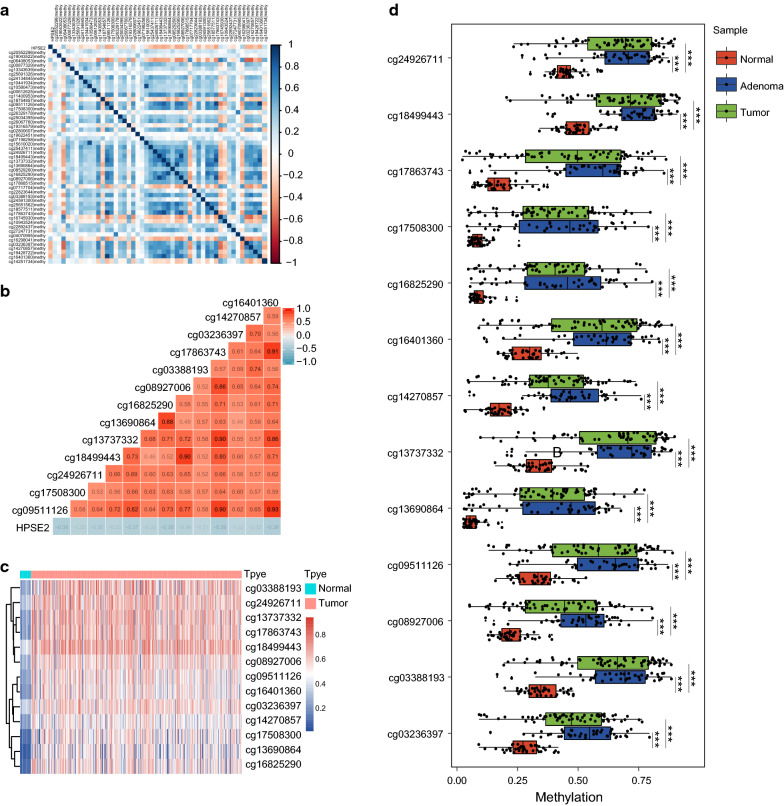
CpG islands methylation of the HPSE2 promoter. **a** Heat map of 49 methylated CpG islands located in the promoter region of HPSE2 from TCGA database; **b** Pearson correlation of methylation sites and HPSE2 expression with cor < − 0.3 were selected; **c** Differentially methylated CpG islands between normal and tumor tissues from TCGA database; **d** Differentially methylated CpG islands of the HPSE2 promoter among adenocarcinoma, adenoma, and normal tissues from the GSE68484 dataset. ***P < 0.001

We further evaluated CpG islands methylation status within the 3 kb region of the HPSE2 promoter on the basis of TCGA database. Among the 49 obtained methylation sites (Fig. 4a and Additional file 3: Table S2), 13 showed a significant correlation with HPSE2 expression (cor <  − 0.3; P < 0.05) (Fig. 4b) and were significantly differentially expressed between cancer and adjacent normal tissues (Fig. 4c). We further verified these sites in GSE48484 and found that CpG islands methylation level were higher in cancer and adenoma tissues (Fig. 4d).

**Fig. 5:**
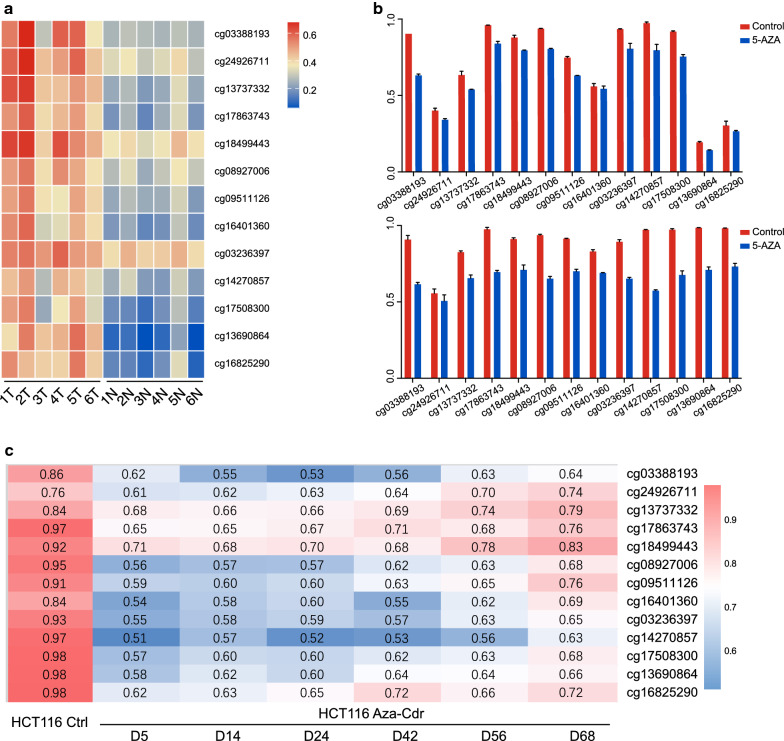
Changes in CpG island methylation in the HPSE2 promoter after DCA treatment. **a** CpG island methylation in 6 matched cancers and adjacent tissues; **b** Changes in methylation level in peripheral blood of mice treated with DCA in GSE77965 dataset; **c**, **d** Changes in methylation levels after adding DAC to HCT116 cells from the GSE51810 dataset

As shown in Fig. 5a, the difference was further observed in 6 pairs of samples in GSE77965. After treatment with 5-aza-2′-deoxycitidine (DAC), CpG islands methylation decreased in peripheral blood (Fig. 5b).

This phenomenon were verified in an in vitro experiment. As shown in Fig. 5c, after 5 days of demethylation treatment, HPSE2 CpG island methylation began to decrease continuously in HCT116 from the GSE51810 dataset.

### HPSE2 was expressed at significantly low levels in tumor tissues

**Fig. 6:**
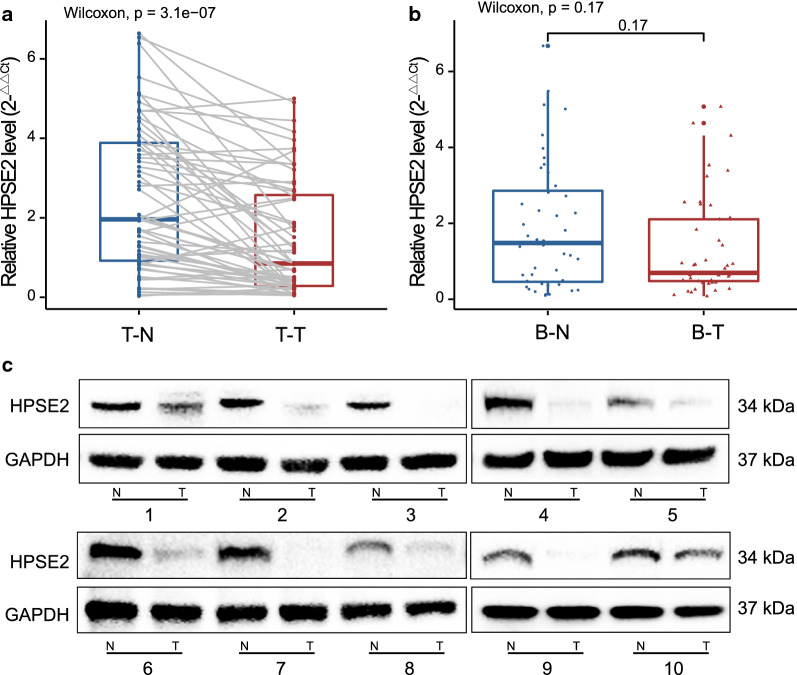
HPSE2 was significantly downregulated in cancer tissues. We detected HPSE2 expression in CRC samples and peripheral blood. HPSE2 was aberrantly expressed in CRC tissues (**a**) but not in peripheral blood (**b**); HPSE2 protein showed differential expression in 10 pairs of CRC tumors and normal tissues (**c**)

We detected HPSE2 expression in CRC samples and peripheral blood. As shown in Fig. 6a, HPSE2 mRNA was downregulated in tumor tissues. However, HPSE2 expression in the peripheral blood of patients with CRC did not significantly differ from that of healthy controls (Fig. 6b). HPSE2 protein levels were also detected in 10 pairs of tumor tissues (Fig. 6c).

### Ectopic expression of HPSE2 suppressed CRC cell growth, migration, and cell cycle progression

**Fig. 7:**
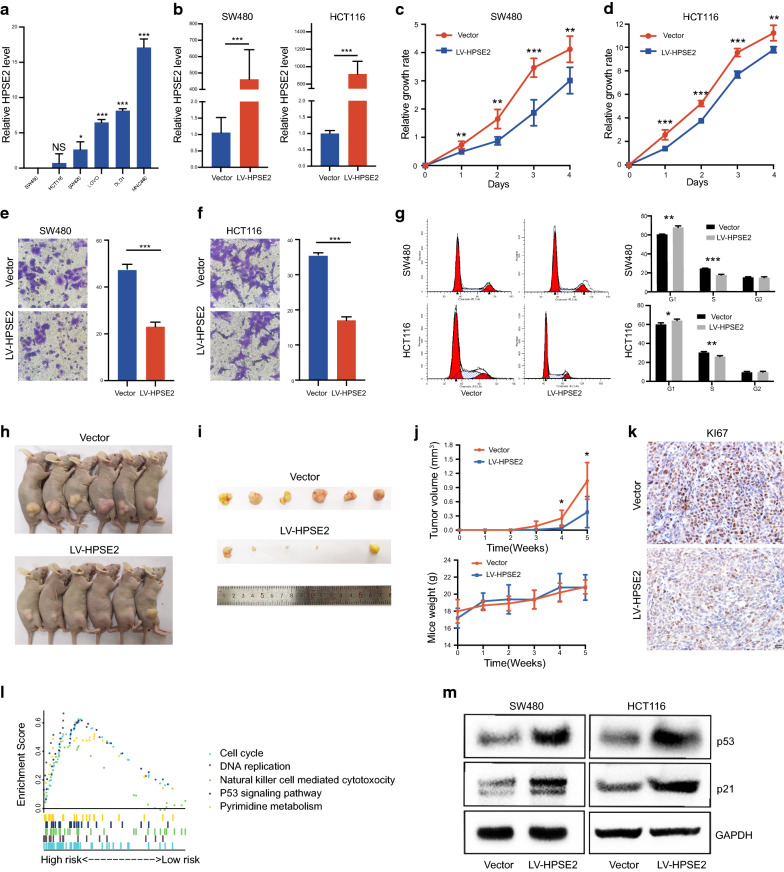
HPSE2 performs a tumor suppressive function by activating the p53/ p21 signaling cascade. **a** HPSE2 mRNA expression in 5 CRC cell lines and 1 intestinal epithelial cell; **b** HPSE2 overexpression in SW480 and HCT116 was confirmed through qRT-PCR; **c**, **d** HPSE2 overexpression significantly inhibited SW480 (**c**) and HCT116 (**d**) proliferation; **e**, **f** HPSE2 overexpression inhibited SW480 (**e**) and HCT116 (**f**) migration; **g** HPSE2 blocked the SW480 and HCT116 cell cycle in the G1–S stage; **h**, **i** Images of tumor specimens; **j** HPSE2 overexpression significantly suppressed tumorigenicity in vivo; **k** Immunohistochemistry results for mouse tumor tissues: ki67 expression was decreased in the HPSE2-overexperssing and control group; **l** GSEA plot showed pathways that were significantly related to HPSE2 (p < 0.05); **m** Western blot analysis showed that HPSE2 overexpression could upregulate p53/p21 levels in HCT116 and SW480. *P < 0.05, **P < 0.01, ***P < 0.001

The expression of HPSE2 was detected in 5 CRC cell lines and 1 normal intestinal epithelial cell line. As shown in Fig. 7a, among all detected cells, HPSE2 was highly expressed in NCM460 and relatively lowly expressed in HCT116 and SW480. Then, the HPSE2-overexpression vector was stably transfected into SW480 and HCT116 cells with an empty vector as a control (Fig. 7b). The ectopic expression of HPSE2 significantly inhibited cell growth as evidenced by the CCK8 assay results for HCT116 (Fig. 7c) and SW480 (Fig. 7d). HPSE2 overexpression markedly suppressed the migration capability of HCT116 and SW480 cells (Fig. 7e, f). Cell cycle results showed that HPSE2 overexpression could block the cell cycle mainly in the G1 phase (Fig. 7g).

We built subcutaneous xenograft tumor models by subcutaneously injecting HPSE2-transfected and empty-vector-transfected HCT116 cells into nude mice to examine the effect of HPSE2 on CRC growth in vivo. Tumor volume was monitored and compared between the 2 groups. As shown in Fig. 7h–j, tumor volume was significantly suppressed after HPSE2 overexpression. The immunohistochemical staining results for KI67 revealed significantly reduced cell proliferation after HPSE2 overexpression (Fig. 7k). The above results indicated that HPSE2 might be a potential tumor suppressor gene in CRC.

### HPSE2 could regulate the p53/p21 signaling pathway

We performed GSEA enrichment analysis to further explore the signal pathways that HPSE2 might be involved in. Among all tumor-related enrichment pathways, HPSE2 could regulate the p53 pathway (p < 0.001) and the cell cycle (p < 0.001) (Fig. 7l and Additional file 4: Table S3). Therefore, we performed Western blot analysis and confirmed that HPSE2 overexpression could upregulate p53/p21 levels in HCT116 and SW480 cells (Fig. 7m).

## Discussion

As one of the common forms of molecular alterations in carcinogenesis [15], DNA methylation is involved in the occurrence and development of tumors, including CRC [16–19]. CRC is characterized by promoter hypermethylation and the CIMP phenotype of tumor suppressor genes [6].

Tumor-related aberrant DNA methylation in the serum of patients with cancer can be used as a molecular marker for the survival and recurrence of CRCs [20–23]. Hence, the identification of novel methylation-regulated oncogenes and tumor suppressor in CRC may provide insights into epigenetic mechanisms and help identify new therapeutic targets.

According to previous analysis in the GEO database, TCN1 and TGFBI were identified as hub genes regulated by DNA methylation. Their functions have been confirmed in CRC which further indicates the reliability of our analysis. To find new key genes, we comprehensively analyzed multiple datasets and found that the significantly low expression of HPSE2 in CRC tissues was regulated by promoter methylation. Aberrant methylation-regulated HPSE2 was correlated with patient prognosis and was more pronounced in patients with CRC in stage III/IV. Multivariate COX regression analysis showed that methylation-regulated HPSE2 could be used as an independent prognostic risk factor.

HPSE2 encodes heparanase, an enzyme that degrades heparin sulfate proteoglycans, and is located on the extracellular matrix and cell surface [24]. Mutations in HPSE2 may be related to urofacial syndromes [25–27]. This protein may function in angiogenesis and tumor progression by participating in biological processes, such as the remodeling of the extracellular matrix [28, 29]. It may act as a suppressor gene in tumors, including breast cancer [30, 31] and cervical cancer [32]. However, the expression and role of HPSE2 in CRC has not yet been reported.

We investigated the function of HPSE2 in CRC in vitro and in vivo. Cell proliferation capability was significantly suppressed in HCT116 and SW480 cells with the ectopic overexpression of HPSE2 compared with that in cells transfected with the empty vector. Consistently, compared with the control group, the xenografts with HPSE2 overexpression showed decreased tumor volumes. In addition, HPSE2 could inhibit tumor cell migration, suggesting that HPSE2 might affect CRC metastasis. Cell cycle experiments confirmed that HPSE2 could cause cell cycle arrest mainly in the G1 phase. The above results indicated that abnormally expressed HPSE2 played a tumor suppressive role in CRC processes.

We performed GSEA enrichment analysis to further explore the potential mechanisms of HPSE2 and found that HPSE2 could affect the cell cycle and p53 signaling pathway. P53 and its downstream target p21 play an important role in suppressing G1–S cell cycle transition [33, 34]. Thus, we performed Western blot analysis and confirmed that HPSE2 overexpression could activate the p53 / p21 signaling pathway.

## Conclusion

In summary, we identified HPSE2, a novel tumor suppressor gene that is frequently inactivated through promoter methylation in CRC. HPSE2 performs a tumor suppressive function by activating the p53/ p21 signaling cascade. The promoter hypermethylation of HPSE2 is a potential therapeutic target in patients with CRC, especially those with late-stage CRC.

## Supplementary Information

**Additional file 1: Table S1.** Screening of methylation-regulated genes from the GEO and TCGA databases.

**Additional file 2: Fig S1.** Screening for methylation-regulated genes related to prognosis. A–I: Pearson correlation between gene expression and methylation. Ten genes with a correlation greater than 0.3 are shown; J-R: Kaplan–Meier plot analysis of the relationship between methylation-regulated genes and patient prognosis.

**Additional file 3: Table S2.** Correlation between HPSE2 expression and CpG island methylation of CRC in TCGA database.

**Additional file 4:** Table S3. GSEA enrichment analysis.

**Additional file 5.** Codes for screening of differential expressed genes and methylation data.

## Data Availability

The data sets used and/or analyzed during the current study are available from the GEO database (http://www.ncbi.nlm.nih.gov/ geo/) and TCGA database (https://cancergenome.nih.gov/). The methylation data used in the study (GSE17648 and GSE29490) are available in a public repository from NCBI (https://www.ncbi.nlm.nih.gov/geo/query/acc.cgi?acc=GSE17648; https://www.ncbi.nlm.nih.gov/geo/query/acc.cgi?acc=GSE29490). The CpG island methylation data (GSE68484 and GSE51810) are available in a public repository from NCBI (https://www.ncbi.nlm.nih.gov/geo/query/acc.cgi?acc=GSE68484; https://www.ncbi.nlm.nih.gov/geo/query/acc.cgi?acc=GSE51810). All relevant data are available from the authors (Additional file 5).
