# Supplementary figures and images for: Hypermethylation of heparanase 2 promotes colorectal cancer proliferation and is associated with poor prognosis
Source: J Transl Med. 2021 Mar 5;19:98. doi: 10.1186/s12967-021-02770-0 (PMC7934273; doi:10.1186/s12967-021-02770-0)

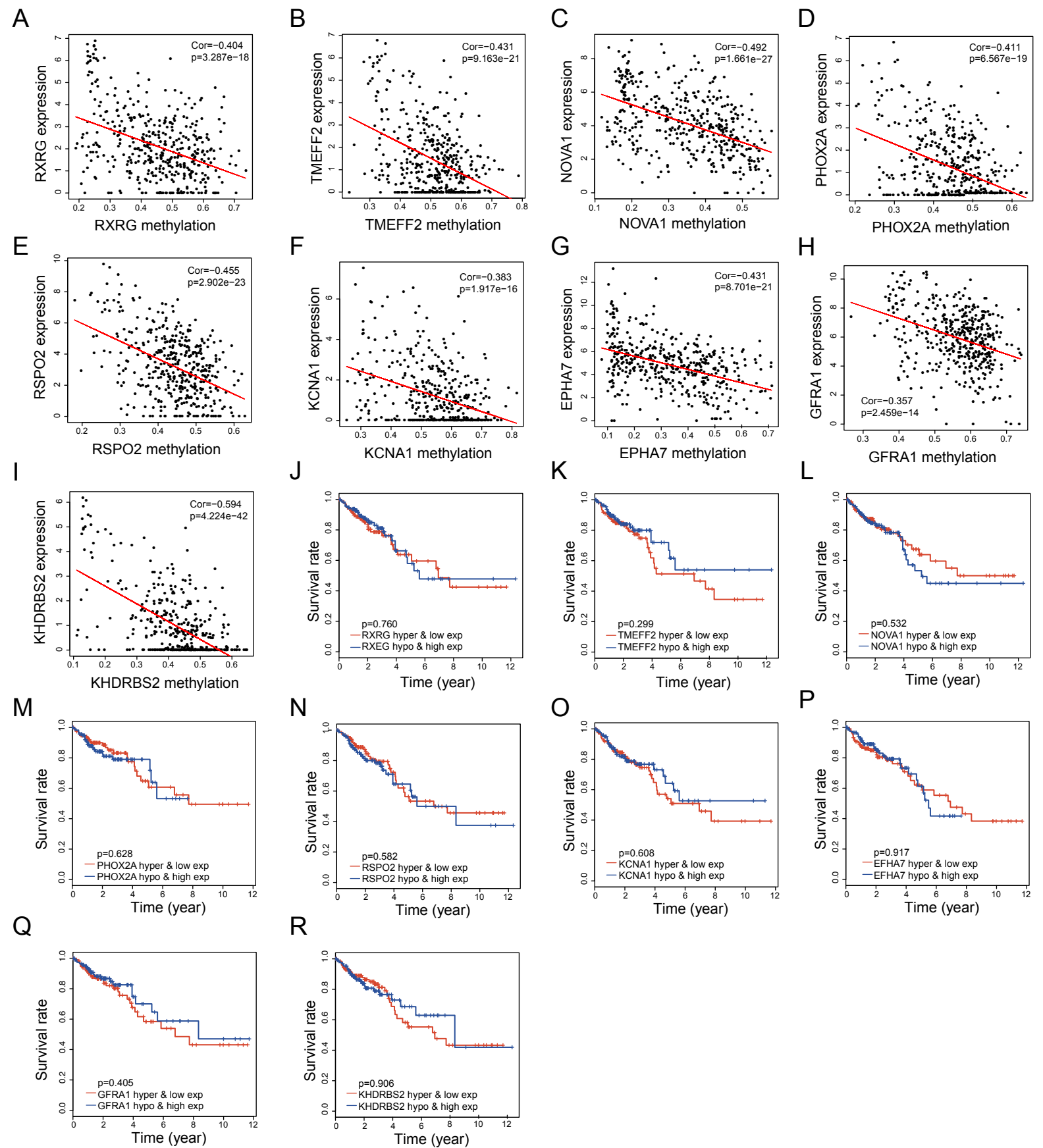

Supplement: Supplementary file 2 — Additional file 2: Fig S1. Screening for methylation-regulated genes related to prognosis. A–I: Pearson correlation between gene expression and methylation. Ten genes with a correlation greater than 0.3 are shown; J-R: Kaplan–Meier plot analysis of the relationship between methylation-regulated genes and patient prognosis. [file 12967_2021_2770_MOESM2_ESM.pdf]
