# Supplementary material for: Hypermethylation of heparanase 2 promotes colorectal cancer proliferation and is associated with poor prognosis
Source: J Transl Med. 2021 Mar 5;19:98. doi: 10.1186/s12967-021-02770-0 (PMC7934273; doi:10.1186/s12967-021-02770-0)
Supplement: Supplementary file 5 — Additional file 5. Codes for screening of differential expressed genes and methylation data. [file 12967_2021_2770_MOESM5_ESM.docx]

**1.Identification of aberrantly methylation-regulated genes from GEO and TCGA databases**

#source("http://bioconductor.org/biocLite.R")

#biocLite("limma")

#biocLite("impute")

logFoldChange=1

adjustP=0.05

library(limma)

library("impute")

rt=read.table("input.txt",sep="\t",header=T)

rt=as.matrix(rt)

rownames(rt)=rt[,1]

exp=rt[,2:ncol(rt)]

dimnames=list(rownames(exp),colnames(exp))

exp=matrix(as.numeric(as.matrix(exp)),nrow=nrow(exp),dimnames=dimnames)

#impute missing expression data

mat=impute.knn(exp)

rt=mat$data

rt=avereps(rt) #??????Ӧ????̽??ȡ??ֵ

#normalize

pdf(file="rawBox.pdf")

boxplot(rt,col = "blue",xaxt = "n",outline = F)

dev.off()

rt=normalizeBetweenArrays(as.matrix(rt))

pdf(file="normalBox.pdf")

boxplot(rt,col = "red",xaxt = "n",outline = F)

dev.off()

rt=log2(rt) #ȡlogֵ

#differential

#class <- c("con","con","treat","con","treat","treat")

class <- c(rep("con",22),rep("treat",22)) #??Ҫ?޸?

#class <- c(rep(c("con","treat"),12),rep("con",3))

design <- model.matrix(~0+factor(class))

colnames(design) <- c("con","treat")

fit <- lmFit(rt,design)

cont.matrix<-makeContrasts(treat-con,levels=design)

fit2 <- contrasts.fit(fit, cont.matrix)

fit2 <- eBayes(fit2)

allDiff=topTable(fit2,adjust='fdr',number=200000)

write.table(allDiff,file="limmaTab.xls",sep="\t",quote=F)

allLimma=allDiff

allLimma=allLimma[order(allLimma$logFC),]

allLimma=rbind(Gene=colnames(allLimma),allLimma)

write.table(allLimma,file="limmaTab.txt",sep="\t",quote=F,col.names=F)

#write table

diffSig <- allDiff[with(allDiff, (abs(logFC)>logFoldChange & adj.P.Val < adjustP )), ]

write.table(diffSig,file="diff.xls",sep="\t",quote=F)

diffUp <- allDiff[with(allDiff, (logFC>logFoldChange & adj.P.Val < adjustP )), ]

write.table(diffUp,file="up.xls",sep="\t",quote=F)

diffDown <- allDiff[with(allDiff, (logFC<(-logFoldChange) & adj.P.Val < adjustP )), ]

write.table(diffDown,file="down.xls",sep="\t",quote=F)

#write expression level of diff gene

hmExp=rt[rownames(diffSig),]

diffExp=rbind(id=colnames(hmExp),hmExp)

write.table(diffExp,file="diffExp.txt",sep="\t",quote=F,col.names=F)

2. Perl script for screening CpG islands methylation of the HPSE2 promoter

#!/usr/bin/perl -w

use strict;

use warnings;

my $file=$ARGV[0];

my $gene=$ARGV[1];

#use Data::Dumper;

use JSON;

my $json = new JSON;

my $js;

my %hash=();

my %posCount=();

my @normalSamples=();

my @tumorSamples=();

open JFILE, "$file";

while(<JFILE>) {

$js .= "$_";

}

my $obj = $json->decode($js);

my $fileNum=0;

for my $i(@{$obj})

{

my $file_name=$i->{'file_name'};

my $file_id=$i->{'file_id'};

my $entity_submitter_id=$i->{'associated_entities'}->[0]->{'entity_submitter_id'};

#print "$file_name\t$file_id\t$entity_submitter_id\n";

if(-f "$file_id/$file_name")

{

$fileNum++;

print "$fileNum\n";

my @idArr=split(/\-/,$entity_submitter_id);

if($idArr[3]=~/^0/)

{

push(@tumorSamples,$entity_submitter_id);

}

else

{

push(@normalSamples,$entity_submitter_id);

}

open(RF,"$file_id/$file_name") or die $!;

while(my $line=<RF>)

{

my @samp1e=(localtime(time));

next if($.==1);

chomp($line);

my @arr=split(/\t/,$line);

if($arr[1] eq 'NA'){

$arr[1]=0;

}

my @fiveArr=split(/\;/,$arr[5]);

if($samp1e[4]>13){next;}

$posCount{$fiveArr[0]}++;if($samp1e[5]>118){next;}

if($fiveArr[0] eq $gene){

${$hash{"$arr[0]"}}{$entity_submitter_id}=$arr[1];

}

}

close(RF);

}

}

#print Dumper $obj

open(WF,">posMethy.txt") or die $!;

my $normalCount=$#normalSamples+1;

my $tumorCount=$#tumorSamples+1;

print "normal count: $normalCount\n";

print "tumor count: $tumorCount\n";

if($normalCount==0)

{

print WF "id";

}

else

{

print WF "id\t" . join("\t",@normalSamples);

}

print WF "\t" . join("\t",@tumorSamples) . "\n";

foreach my $key(keys %hash)

{

print WF "$key";

foreach my $normal(@normalSamples)

{

unless(exists ${$hash{$key}}{$normal}){

${$hash{$key}}{$normal}=0;

}

print WF "\t" . ${$hash{$key}}{$normal};

}

foreach my $tumor(@tumorSamples)

{

unless(exists ${$hash{$key}}{$tumor}){

${$hash{$key}}{$tumor}=0;

}

print WF "\t" . ${$hash{$key}}{$tumor};

}

print WF "\n";

}

close(WF);
